# Adenovirus nephritis in adult kidney allograft recipients: a systematic review of literature

**DOI:** 10.1007/s15010-024-02455-y

**Published:** 2025-01-02

**Authors:** Pothumarthy Venkata Swathi Kiran, Nitin Gupta, Attur Ravindra Prabhu, Anjely Sebastian, Carl Boodman, Tirlangi Praveen Kumar

**Affiliations:** 1 Department of Infectious Diseases, Kasturba Medical College, Manipal, Manipal Academy of Higher Education, Manipal, 576104 India; 2 Department of Nephrology, Kasturba Medical College, Manipal, Manipal Academy of Higher Education, Manipal, 576104 India; 3 Institute of Tropical Medicine, Antwerp, 2000 Belgium; 4 University of Antwerp, Antwerp, Belgium

**Keywords:** Adenovirus, Nephritis, Kidney transplant

## Abstract

**Background:**

Adenovirus nephritis is an increasingly recognized complication in adult kidney transplant recipients, characterized by its diverse clinical presentations and diagnostic challenges. This systematic review summarises the clinical profiles and outcomes of adenoviral nephritis in kidney allograft recipients.

**Methods:**

We conducted a systematic search of PubMed, Embase, and Web of Science for studies (case reports or series) with individual patient data on adult kidney transplant recipients with confirmed or presumptive adenoviral nephritis up to October 2, 2024. Clinical profile, treatment and outcome data with adenoviral nephritis were collected and summarised for all patients. We compared features of early and late adenoviral nephritis (diagnosis before and after 90 days post-transplantation).

**Results:**

Thirty-nine studies met inclusion criteria, involving 57 patients with a mean age of 45.7 years and a male predominance. The median time to infection post-transplant was 168 days. The most common symptoms were fever (68.5%), dysuria (49%) and diarrhoea (21%). Early adenoviral infection was more common in cadaveric graft recipients. Fever and gross haematuria were more common in late adenoviral infections. Biopsies showed interstitial nephritis (100%), with some having acute tubular necrosis (53%). Granulomas were seen in 61.2%. Glomeruli and peritubular capillaries were not affected in any of the biopsies. Reversible graft dysfunction was observed in 75% of cases, while mortality was noted in three patients.

**Conclusion:**

Adenoviral nephritis is associated with diverse clinical manifestations with differing chronology post-transplantation. Graft dysfunction is associated with reversible interstitial nephritis. Further research is necessary to improve outcomes.

**Supplementary Information:**

The online version contains supplementary material available at 10.1007/s15010-024-02455-y.

## Introduction

Adenovirus, an unencapsulated, double-stranded DNA virus, causes a spectrum of infections that can range from asymptomatic cases to severe, life-threatening illnesses, particularly among those with compromised immune systems [1–3]. The manifestations of adenoviral infections are diverse, encompassing various organ systems, including the central nervous system, respiratory tract, gastrointestinal system and the kidneys [1–3]. Adenoviral infections are an increasingly recognised complication in renal allograft recipients, driven by the unique vulnerabilities of this patient population [4, 5]. Reactivation of latent infections is a common pathway for initial infections, while primary infections may emerge later [6]. Haemorrhagic cystitis and nephritis are common manifestations of urinary system involvement in adenovirus infection among renal transplant recipients. Isolated case reports/ series describe various biopsy findings in adenoviral nephritis, such as tubulointerstitial nephritis, granulomas, and necrosis [7]. While adenoviral haemorrhagic cystitis is well described, adenoviral nephritis is a poorly characterized entity [8]. Therefore, we did a systematic review to elucidate the clinical features, diagnostic modalities, and management strategies for adenoviral nephritis in kidney allograft recipients.

## Methodology

### Registration

The review followed the Preferred Reporting Items for Systematic Reviews and Meta-Analyses (PRISMA) guidelines for systematic literature reviews and was registered in the International Prospective Register of Systematic Reviews (PROSPERO CRD42024597199).

### Research Question

In patients with renal transplants, what are the clinical manifestations, laboratory findings and outcomes of patients with adenoviral nephritis?

### Search strategy

A systematic search of PubMed, Embase and Web of Science was conducted to identify studies with individual case details on Adenoviral nephritis in adult renal allograft recipients. The search aimed to capture all relevant articles up until 2nd October 2024, using the following keywords: (adenovirus OR adenoviral) AND (transplant) AND (nephropathy OR nephritis OR kidney OR renal).

### Eligibility criteria

Studies of adult kidney transplant recipients (aged > 18 years) diagnosed with confirmed or presumptive adenovirus nephritis were included. Those with a positive immunohistochemistry (IHC) or polymerase chain reaction (PCR) on the biopsy were confirmed as adenoviral nephritis. Patients with clinical signs and symptoms, suggestive biopsy findings and concurrent adenovirus viruria or viraemia who did not undergo biopsy were considered presumptive adenoviral nephritis.

### Screening

After deleting the duplicates, two reviewers (PVSK and TPK) independently screened the title and abstract. Full texts of all included articles were retrieved for independent screening by the same two authors. The third reviewer (NG) resolved any discrepancy in both screening phases.

### Critical appraisal

The methodological quality of the included studies was assessed using the Joanna Briggs Institute (JBI) critical appraisal tools for case reports.

### Data extraction

Data were extracted independently by two reviewers (PVSK and TPK) using a standardised form containing study characteristics (author, year of publication), patient demographics (age, gender, underlying diseases), details of adenoviral infection (timing, diagnostic methods), biopsy findings, radiological findings, treatment details and clinical outcomes (mortality, graft loss, reversible dysfunction).

### Data analysis

Qualitative variables were expressed as frequency and percentage. Continuous variables were expressed as mean *±* standard deviation (SD) or median with interquartile range (in brackets). The patients were classified as early or late adenoviral infections (before or after 90 days of kidney transplantation, respectively). Clinical features and outcomes were compared between early vs. late infections. Factors associated with reversible vs. irreversible graft dysfunctions were also compared. The chi-square test, t-test or Mann-Whitney U test were used as the statistical tests of significance. A p-value of < 0.05 was considered as significant. Rayyan.ai was used for screening, and IBM SPSS software (version 26) was used for statistical analysis.

## Results

### Selection of studies

**Fig. 1:**
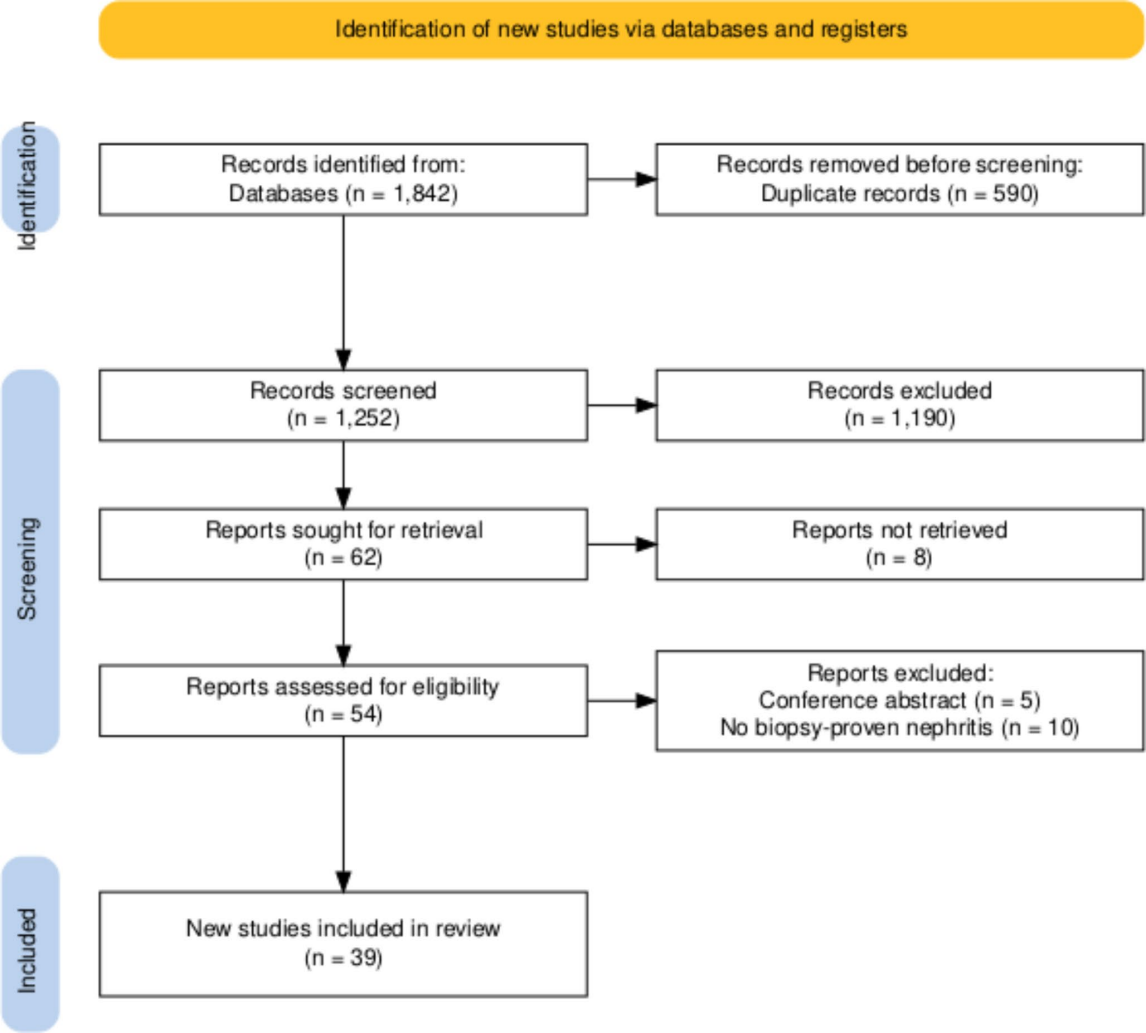
PRISMA diagram showing screening and final inclusion of the patients with adenoviral nephritis

Of the 1842 records identified, 590 duplicates were removed (Fig. 1). After screening 1252 titles and abstracts, 1190 records were excluded as they did not meet the eligibility criteria. After initial screening, 62 records were identified as eligible for full-text screening. Of 54 retrieved reports (full text not available-8), 15 were excluded (conference abstracts-5, no nephritis on biopsy = 10), and 39 studies (57 patients) were included for data extraction and analysis (Fig. 1).

### Demographic profile of included patients

The mean age of the 57 included patients was 45.7 ± 14.4 years, with a predominance of male patients, who constituted 74% (*n* = 42) of the population. The median time to infection following transplantation was 168 days (IQR: 30–525 days), with 47% (*n* = 27) classified as early and the remaining 53% (*n* = 30) as late. Most donors were deceased/cadaveric (68%, 36/53). Induction with IL-2 blockers was utilised in 49% (20/41) of cases, while antithymocyte globulin (ATG) was administered in 39% (16/41). Apart from steroids, tacrolimus (91%, 49/55), mycophenolate mofetil (91%, 50/55), mTOR inhibitors (9%, 5/55) and cyclosporine (4%, 2/55) were used in various combinations for maintenance immunosuppression (Table 1).

**Table 1:** Demographic characteristics of patients with adenoviral nephritis

Sn	Authors	Age (y)	Sex	Time to infection (days)	Early vs. Late	Donor	IL-2 RA	ATG	Tac	CyS	MMF	AZA	m TOR
1	Sujeet et al. [8]	65	F	1095	Late	Cadaveric	NA	NA	Yes	No	Yes	No	No
2	Rady et al. [9]	63	M	42	Early	Cadaveric	Yes	No	Yes	No	Yes	No	No
3	Friedrichs et al. [10]	48	F	28	Early	Cadaveric	Yes	No	Yes	No	Yes	No	No
4	Harshavardhan et al. [11]	45	M	28	Early	Cadaveric	No	Yes	Yes	No	Yes	No	No
5	Seralathan et al. [12]	37	M	23	Early	Cadaveric	Yes	No	Yes	No	Yes	No	No
6	Watanabe et al. [13]	52	F	630	Late	Live	Yes	No	Yes	No	Yes	No	No
7	Cullen et al. [14]	51	M	48	Early	NA	NA	NA	Yes	No	Yes	No	No
8	Cullen et al. [14]	55	M	660	Late	NA	NA	NA	NA	NA	NA	NA	NA
9	Cullen et al. [14]	61	F	42	Early	NA	NA	NA	NA	NA	NA	NA	NA
10	Fontalvo et al. [15]	40	F	70	Early	Cadaveric	No	No	Yes	No	No	No	Yes
11	Lum et al. [16]	30	M	480	Late	Cadaveric	Yes	No	Yes	No	Yes	No	No
12	Bruminhent et al. [6]	34	M	30	Early	Cadaveric	No	Yes	Yes	No	Yes	No	Yes
13	Bruminhent et al. [6]	53	M	360	Late	Cadaveric	Yes	No	Yes	No	Yes	No	No
14	Attieh et al. [17]	35	M	8	Early	Cadaveric	No	Yes	Yes	No	Yes	No	No
15	Jagannatham et al. [18]	29	M	72	Early	Cadaveric	No	Yes	Yes	No	Yes	No	No
16	Jagannatham et al. [18]	44	M	1443	Late	Live	No	No	Yes	No	Yes	No	No
17	Jagannatham et al. [18]	76	M	257	Late	Cadaveric	Yes	No	No	No	No	No	No
18	Jagannatham et al. [18]	18	M	32	Early	Cadaveric	No	Yes	Yes	No	Yes	No	No
19	Jagannatham et al. [18]	60	M	692	Late	Cadaveric	No	Yes	Yes	No	No	No	No
20	Jagannatham et al. [18]	50	M	1154	Late	Cadaveric	NA	NA	Yes	No	No	No	No
21	Jagannatham et al. [18]	39	F	124	Late	Live	No	Yes	Yes	No	Yes	No	No
22	Jagannatham et al. [18]	22	M	931	Late	Cadaveric	No	Yes	Yes	No	Yes	No	No
23	Jagannatham et al. [18]	50	M	4582	Late	Cadaveric	No	Yes	Yes	No	Yes	No	No
24	Jagannatham et al. [18]	34	M	671	Late	Cadaveric	Yes	No	No	No	Yes	No	Yes
25	Jagannatham et al. [18]	57	M	240	Late	Cadaveric	Yes	No	Yes	No	Yes	No	No
26	Fujita et al. [19]	40	M	1642	Late	Live	Yes	No	Yes	No	Yes	No	No
27	Pochineni et al. [20]	75	F	90	Early	Cadaveric	No	Yes	Yes	No	Yes	No	No
28	Lucia et al. [21]	39	M	300	Late	Live	NA	NA	Yes	No	Yes	No	No
29	Alquadan et al. [22]	33	F	480	Late	Cadaveric	NA	NA	Yes	No	Yes	No	No
30	Thorne et al. [23]	61	M	210	Late	Cadaveric	NA	NA	NA	No	Yes	No	No
31	Moreira et al. [24]	40	M	17	Early	Live	Yes	No	Yes	No	Yes	No	No
32	Silva et al. [25]	38	M	540	Late	Live	NA	NA	Yes	No	Yes	No	No
33	Veer et al. [26]	75	F	365	Late	Live	Yes	No	Yes	No	Yes	No	No
34	Nanmoku et al. [27]	57	M	168	Late	Live	NA	NA	Yes	No	Yes	No	No
35	Nanmoku et al. [27]	39	M	183	Late	Live	NA	NA	Yes	No	Yes	No	No
36	Nanmoku et al. [27]	33	M	413	Late	Live	NA	NA	Yes	No	Yes	No	No
37	Nanmoku et al. [27]	39	M	325	Late	Live	NA	NA	Yes	No	Yes	No	No
38	Nanmoku et al. [27]	36	M	1763	Late	Live	NA	NA	Yes	No	Yes	No	No
39	Park et al. [28]	32	F	300	Late	Cadaveric	Yes	No	Yes	No	Yes	No	No
40	Saliba et al. [29]	41	M	14	Early	Cadaveric	No	Yes	Yes	No	Yes	No	No
41	Ramirez et al. [30]	27	M	30	Early	Live	Yes	No	Yes	No	Yes	No	No
42	Parasuraman et al. [31]	44	F	22	Early	Cadaveric	Yes	No	Yes	No	Yes	No	No
43	Joyon et al. [32]	56	F	42	Early	Cadaveric	No	Yes	Yes	No	Yes	No	No
44	Storsley et al. [33]	54	M	42	Early	Live	No	No	Yes	No	Yes	No	No
45	Varma et al. [34]	57	M	25	Early	Cadaveric	No	Yes	Yes	No	Yes	No	No
46	Kozlowski et al. [35]	44	M	17	Early	Cadaveric	No	Yes	Yes	No	Yes	No	No
47	Kozlowski et al. [35]	55	M	43	Early		No	No	Yes	No	Yes	No	No
48	Kolankiewicz et al. [36]	45	F	510	Late	Cadaveric	No	Yes	Yes	No	Yes	No	No
49	Barraclough et al. [37]	68	M	14	Early	Cadaveric	Yes	No	Yes	No	Yes	No	No
50	Paula et al. [38]	32	M	30	Early	Cadaveric	Yes	No	Yes	No	No	No	Yes
51	Hensley et al. [39]	27	F	180	Late	Live	No	Yes	Yes	No	Yes	No	No
52	Gaspert et al. [40]	64	M	60	Early	Cadaveric	NA	NA	No	Yes	Yes	No	No
53	Alsaad et al. [41]	19	M	4380	Late	Cadaveric	NA	NA	Yes	No	Yes	No	No
54	Rosario et al. [42]	58	M	22	Early	Cadaveric	Yes	No	No	No	Yes	No	Yes
55	Lim et al. [43]	51	M	36	Early	Cadaveric	Yes	No	Yes	No	Yes	No	No
56	Asim et al. [44]	60	M	27	Early	Live	Yes	No	No	Yes	Yes	No	No
57	Lachiewicz et al. [45]	20	F	600	Late	Cadaveric	No	No	Yes	No	Yes	No	No

Abbreviations: Sn- Serial number, y- years, Male, F-Female, IL-2RA-Interleukin-2 receptor blockers; ATG-Antithymocyte globulin; Tac- Tacrolimus; CyS- cyclosporine; MMF- mycophenolate mofetil; AZA- Azathioprine; mTOR inhibitor-mammalian target of rapamycin, NA- Data not available

### Clinical profile

The most commonly reported symptoms were fever (68.5%, 37/54), dysuria (49%, 27/55), and diarrhoea (21%, 12/56). Gross/macroscopic haematuria was seen in 51% (29/57), and microscopic haematuria was seen in 80% (43/54) (Table 2). Except for two patients, all had graft dysfunction (as determined by the clinical team) at the time of diagnosis. Radiological evaluation revealed hydronephrosis in four cases and multiple cortical hypodensities in six cases. Coexistent viremia was noted in 94% (34 out of 36) of patients, while viruria was present in 97% (33 out of 34) of cases.

**Table 2:** Clinical features of patients with adenoviral nephritis

Sn	Authors	Fever	Dysuria	Diarrhea	Macroscopic hematuria	Microscopic hematuria	Graft dysfunction	Viremia	Viruria
1	Sujeet et al. [9]	NA	No	No	Yes	Yes	Yes	Yes	Yes
2	Rady et al. [10]	Yes	Yes	Yes	Yes	Yes	Yes	Yes	Yes
3	Friedrichs et al. [11]	No	No	No	No	Yes	Yes	Yes	Yes
4	Harshavardhan et al. [12]	Yes	No	No	No	Yes	Yes	Yes	Yes
5	Seralathan et al. [13]	No	No	No	Yes	Yes	Yes	NA	Yes
6	Watanabe et al. [14]	Yes	No	No	No	No	No	Yes	Yes
7	Cullen et al. [15]	No	Yes	No	Yes	Yes	Yes	No	Yes
8	Cullen et al. [15]	Yes	Yes	Yes	Yes	Yes	Yes	Yes	Yes
9	Cullen et al. [15]	Yes	Yes	Yes	No	NA	Yes	NA	NA
10	Fontalvo et al. [16]	No	No	No	No	No	Yes	Yes	NA
11	Lum et al. [17]	Yes	No	No	Yes	Yes	Yes	NA	Yes
12	Bruminhent et al. [6]	NA	NA	No	Yes	Yes	Yes	Yes	Yes
13	Bruminhent et al. [6]	NA	NA	NA	Yes	NA	Yes	Yes	Yes
14	Attieh et al. [18]	Yes	No	Yes	No	NA	Yes	Yes	Yes
15	Jagannatham et al. [7]	Yes	Yes	Yes	Yes	Yes	Yes	Yes	Yes
16	Jagannatham et al. [7]	Yes	No	No	No	Yes	Yes	Yes	Yes
17	Jagannatham et al. [7]	Yes	No	No	No	No	Yes	Yes	Yes
18	Jagannatham et al. [7]	No	No	No	No	No	Yes	Yes	Yes
19	Jagannatham et al. [7]	Yes	No	No	Yes	Yes	Yes	Yes	Yes
20	Jagannatham et al. [7]	Yes	No	No	No	Yes	Yes	NA	NA
21	Jagannatham et al. [7]	No	Yes	No	No	Yes	Yes	Yes	NA
22	Jagannatham et al. [7]	Yes	Yes	Yes	No	Yes	Yes	NA	Yes
23	Jagannatham et al. [7]	No	No	No	No	Yes	Yes	NA	NA
24	Jagannatham et al. [7]	Yes	Yes	Yes	Yes	Yes	Yes	NA	NA
25	Jagannatham et al. [7]	Yes	No	No	No	Yes	Yes	NA	NA
26	Fujita et al. [19]	Yes	Yes	No	Yes	Yes	Yes	NA	Yes
27	Pochineni et al. [20]	No	Yes	Yes	Yes	Yes	Yes	Yes	Yes
28	Lucia et al. [21]	Yes	Yes	No	No	Yes	Yes	NA	Yes
29	Alquadan et al. [22]	Yes	No	No	Yes	Yes	Yes	Yes	Yes
30	Thorne et al. [23]	Yes	Yes	No	Yes	Yes	Yes	NA	NA
31	Moreira et al. [24]	Yes	No	Yes	No	Yes	Yes	Yes	Yes
32	Silva et al. [25]	Yes	Yes	No	Yes	Yes	Yes	NA	NA
33	Veer et al. [26]	Yes	Yes	Yes	Yes	Yes	Yes	Yes	NA
34	Nanmoku et al. [27]	No	Yes	No	Yes	Yes	Yes	Yes	NA
35	Nanmoku et al. [27]	Yes	Yes	No	Yes	Yes	Yes	Yes	Yes
36	Nanmoku et al. [27]	Yes	Yes	No	Yes	Yes	Yes	Yes	Yes
37	Nanmoku et al. [27]	Yes	Yes	No	Yes	Yes	Yes	NA	Yes
38	Nanmoku et al. [27]	Yes	Yes	No	Yes	Yes	Yes	Yes	Yes
39	Park et al. [28]	Yes	Yes	No	Yes	Yes	Yes	Yes	Yes
40	Saliba et al. [29]	Yes	Yes	No	No	Yes	Yes	Yes	NA
41	Ramirez et al. [30]	Yes	Yes	No	No	Yes	Yes	Yes	Yes
42	Parasuraman et al. [31]	No	No	Yes	Yes	Yes	Yes	Yes	Yes
43	Joyon et al. [32]	No	No	No	No	No	Yes	NA	NA
44	Storsley et al. [33]	No	No	No	No	No	Yes	NA	NA
45	Varma et al. [34]	Yes	No	No	No	No	Yes	Yes	NA
46	Kozlowski et al. [35]	No	No	No	Yes	Yes	Yes	NA	NA
47	Kozlowski et al. [35]	No	No	No	No	No	Yes	NA	NA
48	Kolankiewicz et al. [36]	Yes	No	No	Yes	Yes	Yes	Yes	NA
49	Barraclough et al. [37]	Yes	Yes	No	Yes	Yes	Yes	Yes	Yes
50	Paula et al. [38]	Yes	Yes	No	No	Yes	Yes	Yes	Yes
51	Hensley et al. [39]	No	No	No	No	No	Yes	NA	NA
52	Gaspert et al. [40]	No	No	No	No	Yes	Yes	NA	NA
53	Alsaad et al. [41]	Yes	Yes	No	Yes	Yes	Yes	NA	NA
54	Rosario et al. [42]	Yes	No	Yes	No	No	Yes	Yes	NA
55	Lim et al. [43]	Yes	Yes	No	No	Yes	Yes	No	No
56	Asim et al. [44]	No	Yes	No	Yes	Yes	Yes	NA	NA
57	Lachiewicz et al. [45]	Yes	No	No	No	No	No	Yes	Yes

### Biopsy findings

Biopsy findings revealed acute tubulointerstitial nephritis in all patients and concomitant acute tubular necrosis (ATN) in 53% (26/49) of patients (Table 3). Additionally, four patients had biopsy findings suggestive of concomitant acute rejection [7, 32, 33]. Viral cytopathic changes were identified in all cases, with immunohistochemistry (IHC) testing positive in 87% (40/46) of instances. Areas of necrosis were present in 40.4% (19/47) patients, whereas haemorrhage in the interstitium was present in 21.2% (10/47). Granulomas were present in 61.2% (30/49) of the patients. Among the 19 patients where granulomas were characterised, 78.9% (15/19) had necrotising granulomas.

**Table 3:** Details of biopsy findings in patients with adenoviral nephritis

Sn	Authors	Mono	PMN	TIN	ATN	Necrosis	Hmg	Granulomas	Viral inclusions	IHC OR Tissue PCR positive
1	Sujeet et al. [9]	Yes	Yes	Yes	Yes	No	No	Yes	Yes	Yes
2	Rady et al. [10]	NA	NA	Yes	No	Yes	No	No	Yes	Yes
3	Friedrichs et al. [11]	Yes	No	Yes	Yes	No	No	No	Yes	Yes
4	Harshavardhan et al. [12]	Yes	Yes	Yes	Yes	Yes	Yes	No	Yes	Yes
5	Seralathan et al. [13]	Yes	Yes	Yes	No	No	Yes	No	Yes	Yes
6	Watanabe et al. [14]	NA	NA	Yes	No	No	Yes	Yes	Yes	No
7	Cullen et al. [15]	Yes	Yes	Yes	Yes	Yes	No	No	Yes	Yes
8	Cullen et al. [15]	Yes	Yes	Yes	Yes	Yes	No	No	Yes	NA
9	Cullen et al. [15]	Yes	Yes	Yes	No	NA	NA	No	Yes	Yes
10	Fontalvo et al. [16]	Yes	No	NA	NA	No	No	Yes	NA	NA
11	Lum et al. [17]	NA	NA	Yes	No	No	No	Yes	Yes	Yes
12	Bruminhent et al. [6]	NA	NA	Yes	No	NA	NA	No	Yes	NA
13	Bruminhent et al. [6]	NA	NA	Yes	No	NA	NA	No	Yes	NA
14	Attieh et al. [18]	NA	NA	Yes	No	No	Yes	NG	Yes	Yes
15	Jagannatham et al. [7]	No	Yes	Yes	Yes	No	Yes	Yes	Yes	Yes
16	Jagannatham et al. [7]	No	Yes	Yes	Yes	No	No	Yes	Yes	Yes
17	Jagannatham et al. [7]	No	Yes	Yes	Yes	No	No	Yes	Yes	Yes
18	Jagannatham et al. [7]	No	Yes	Yes	No	No	No	Yes	Yes	Yes
19	Jagannatham et al. [7]	No	Yes	Yes	No	No	No	Yes	Yes	Yes
20	Jagannatham et al. [7]	No	Yes	Yes	Yes	Yes	No	Yes	Yes	Yes
21	Jagannatham et al. [7]	No	Yes	Yes	No	No	No	No	Yes	Yes
22	Jagannatham et al. [7]	No	Yes	Yes	Yes	No	Yes	Yes	Yes	Yes
23	Jagannatham et al. [7]	No	Yes	Yes	Yes	No	No	Yes	Yes	Yes
24	Jagannatham et al. [7]	No	Yes	Yes	Yes	Yes	No	No	Yes	Yes
25	Jagannatham et al. [7]	No	Yes	Yes	Yes	No	No	Yes	Yes	Yes
26	Fujita et al. [19]	Yes	No	Yes	No	Yes	No	No	Yes	Yes
27	Pochineni et al. [20]	NA	NA	NA	NA	NA	NA	NA	NA	No
28	Lucia et al. [21]	NA	NA	NA	NA	NA	NA	NA	NA	NA
29	Alquadan et al. [22]	Yes	No	Yes	No	No	No	No	Yes	Yes
30	Thorne et al. [23]	NA	NA	Yes	No	Yes	No	NG	Yes	Yes
31	Moreira et al. [24]	Yes	No	Yes	No	No	No	Yes	Yes	Yes
32	Silva et al. [25]	No	Yes	Yes	No	Yes	No	NG	Yes	Yes
33	Veer et al. [26]	Yes	No	Yes	No	Yes	No	Yes	Yes	Yes
34	Nanmoku et al. [27]	Yes	No	Yes	No	No	No	No	Yes	Yes
35	Nanmoku et al. [27]	Yes	No	NA	NA	NA	NA	NA	NA	NA
36	Nanmoku et al. [27]	Yes	No	NA	NA	NA	NA	NA	NA	NA
37	Nanmoku et al. [27]	Yes	No	NA	NA	NA	NA	NA	NA	NA
38	Nanmoku et al. [27]	Yes	No	NA	NA	NA	NA	NA	NA	NA
39	Park et al. [28]	No	Yes	Yes	Yes	No	No	NG	Yes	Yes
40	Saliba et al. [29]	No	Yes	Yes	Yes	Yes	No	No	Yes	Yes
41	Ramirez et al. [30]	Yes	No	Yes	No	No	No	Yes	Yes	No
42	Parasuraman et al. [31]	Yes	Yes	Yes	Yes	No	No	Yes	Yes	No
43	Joyon et al. [32]	No	Yes	Yes	Yes	Yes	No	No	Yes	Yes
44	Storsley et al. [33]	No	Yes	Yes	No	No	No	Yes	Yes	Yes
45	Varma et al. [34]	Yes	Yes	Yes	Yes	No	No	NG	Yes	Yes
46	Kozlowski et al. [35]	No	Yes	Yes	Yes	Yes	Yes	NG	Yes	Yes
47	Kozlowski et al. [35]	Yes	Yes	Yes	Yes	Yes	No	No	Yes	Yes
48	Kolankiewicz et al. [36]	Yes	No	Yes	Yes	Yes	Yes	No	Yes	No
49	Barraclough et al. [37]	NA	NA	Yes	Yes	Yes	No	Yes	Yes	Yes
50	Paula et al. [38]	Yes	No	Yes	Yes	Yes	No	Yes	Yes	NA
51	Hensley et al. [39]	NA	NA	Yes	No	No	Yes	Yes	Yes	Yes
52	Gaspert et al. [40]	Yes	No	Yes	Yes	No	No	Yes	Yes	Yes
53	Alsaad et al. [41]	Yes	Yes	Yes	Yes	Yes	No	Yes	Yes	Yes
54	Rosario et al. [42]	NA	NA	NA	NA	NA	NA	NA	NA	NA
55	Lim et al. [43]	Yes	Yes	Yes	Yes	Yes	No	No	Yes	Yes
56	Asim et al. [44]	Yes	Yes	Yes	No	No	Yes	Yes	Yes	Yes
57	Lachiewicz et al. [45]	No	Yes	Yes	No	No	No	Yes	Yes	No

Mono- Mononuclear infiltrates, PMN- Polymorphonuclear infiltrates; ATN- Acute tubular necrosis; TIN- Tubulointerstitial nephritis, IHC-Immunohistochemistry; PCR- Polymerase chain reaction, Hmg- Haemorrhage

### Treatment

Management of patients with Adenoviral nephritis involved a reduction in immunosuppression for 93% (53 out of 57) of cases. Mycophenolic acid mofetil was discontinued in 44% (11 out of 25). Antiviral therapy with cidofovir was administered to 28% (16/57) of patients, while intravenous immunoglobulins were given to 54% (31/57) of the cases. (Table 4).

### Outcomes

Acute reversible allograft dysfunction was observed in 75% of cases (43/57), while irreversible graft dysfunction occurred in 24.6% (14/57). Graft loss was seen in 14% (8/57) of the patients. Mortality was noted in three patients: one experienced graft loss and sepsis, while the other two had functioning grafts but suffered from disseminated adenovirus infection deemed to be responsible for patient demise (Table 4).

**Table 4:** Treatment and outcomes of patients with adenoviral nephritis

Sn	Authors	Reduction in IS	CNI stopped	MMF stopped	Cidofovir	Cidofovir nephrotoxicity	Probenicid	IVIG	Reversible allograft dysfunction	Graft loss	Death
1	Sujeet et al. [9]	Yes	No	Yes	Yes	Yes	Yes	No	Yes	No	No
2	Rady et al. [10]	Yes	NA	NA	No	NA	No	No	Yes	No	No
3	Friedrichs et al. [11]	Yes	No	Yes	No	NA	No	Yes	Yes	No	No
4	Harshavardhan et al. [12]	Yes	Yes	Yes	No	NA	No	Yes	No	Yes	Yes
5	Seralathan et al. [13]	Yes	No	No	No	NA	No	No	Yes	No	No
6	Watanabe et al. [14]	Yes	No	No	No	NA	No	No	Yes	No	No
7	Cullen et al. [15]	No	No	No	Yes	Yes	NA	Yes	Yes	No	No
8	Cullen et al. [15]	Yes	NA	NA	No	NA	No	No	Yes	No	No
9	Cullen et al. [15]	Yes	NA	NA	Yes	Yes	NA	Yes	Yes	No	No
10	Fontalvo et al. [16]	Yes	NA	NA	No	NA	No	Yes	Yes	No	No
11	Lum et al. [17]	Yes	No	Yes	Yes	NA	Yes	No	Yes	No	No
12	Bruminhent et al. [6]	Yes	NA	NA	Yes	NA	Yes	Yes	No	Yes	No
13	Bruminhent et al. [6]	Yes	NA	NA	Yes	NA	Yes	Yes	No	No	No
14	Attieh et al. [18]	Yes	No	Yes	Yes	NA	Yes	Yes	No	No	No
15	Jagannatham et al. [7]	Yes	NA	NA	No	NA	No	Yes	Yes	No	No
16	Jagannatham et al. [7]	Yes	NA	NA	Yes	NA	NA	Yes	Yes	No	No
17	Jagannatham et al. [7]	Yes	NA	NA	Yes	NA	NA	No	No	No	Yes
18	Jagannatham et al. [7]	Yes	NA	NA	NA	NA	NA	Yes	No	Yes	No
19	Jagannatham et al. [7]	Yes	NA	NA	No	NA	No	Yes	No	Yes	No
20	Jagannatham et al. [7]	Yes	NA	NA	nNo	NA	No	No	No	Yes	No
21	Jagannatham et al. [7]	Yes	NA	NA	NA	NA	NA	Yes	Yes	No	No
22	Jagannatham et al. [7]	Yes	NA	NA	Yes	NA	NA	No	Yes	No	No
23	Jagannatham et al. [7]	Yes	NA	NA	No	NA	No	No	Yes	No	No
24	Jagannatham et al. [7]	Yes	NA	NA	No	NA	No	Yes	Yes	No	No
25	Jagannatham et al. [7]	No	No	No	No	NA	No	Yes	Yes	No	No
26	Fujita et al. [19]	Yes	NA	NA	No	NA	No	Yes	Yes	No	No
27	Pochineni et al. [20]	Yes	NA	NA	Yes	NA	Yes	No	Yes	No	No
28	Lucia et al. [21]	Yes	NA	NA	No	NA	No	No	Yes	No	No
29	Alquadan et al. [22]	Yes	No	Yes	Yes	NA	NA	Yes	Yes	No	No
30	Thorne et al. [23]	Yes	NA	NA	No	NA	No	No	Yes	No	No
31	Moreira et al. [24]	Yes	No	Yes	No	NA	No	Yes	Yes	No	No
32	Silva et al. [25]	Yes	NA	NA	No	NA	No	Yes	Yes	No	No
33	Veer et al. [26]	Yes	NA	NA	No	NA	No	Yes	Yes	No	No
34	Nanmoku et al. [27]	Yes	NA	No	No	NA	No	Yes	Yes	No	No
35	Nanmoku et al. [27]	Yes	No	No	No	NA	No	Yes	Yes	No	No
36	Nanmoku et al. [27]	No	No	No	No	NA	No	No	Yes	No	No
37	Nanmoku et al. [27]	No	No	No	No	NA	No	No	Yes	No	No
38	Nanmoku et al. [27]	Yes	No	No	No	NA	No	No	Yes	No	No
39	Park et al. [28]	Yes	NA	Yes	No	NA	No	Yes	Yes	No	No
40	Saliba et al. [29]	Yes	NA	NA	No	NA	No	No	Yes	No	No
41	Ramirez et al. [30]	Yes	NA	NA	No	NA	No	Yes	Yes	No	No
42	Parasuraman et al. [31]	Yes	No	Yes	Yes	NA	NA	Yes	Yes	No	No
43	Joyon et al. [32]	Yes	No	No	No	NA	No	Yes	Yes	No	No
44	Storsley et al. [33]	Yes	No	Yes	No	NA	No	Yes	No	No	No
45	Varma et al. [34]	Yes	No	No	No	NA	No	Yes	Yes	No	No
46	Kozlowski et al. [35]	Yes	NA	NA	No	NA	No	No	No	Yes	No
47	Kozlowski et al. [35]	Yes	NA	NA	No	NA	No	No	No	Yes	No
48	Kolankiewicz et al. [36]	Yes	NA	NA	No	NA	No	No	Yes	No	No
49	Barraclough et al. [37]	Yes	NA	NA	Yes	NA	NA	Yes	Yes	No	No
50	Paula et al. [38]	Yes	NA	NA	No	NA	No	Yes	Yes	No	No
51	Hensley et al. [39]	Yes	No	Yes	No	NA	No	No	Yes	No	No
52	Gaspert et al. [40]	Yes	NA	NA	No	NA	No	No	No	Yes	No
53	Alsaad et al. [41]	Yes	NA	NA	Yes	NA	NA	No	Yes	No	No
54	Rosario et al. [42]	Yes	No	No	No	NA	No	No	No	No	Yes
55	Lim et al. [43]	Yes	No	No	No	NA	No	No	Yes	No	No
56	Asim et al. [44]	Yes	No	No	No	NA	No	No	Yes	No	No
57	Lachiewicz et al. [45]	Yes	NA	NA	Yes	No	No	Yes	No	No	No

CNI- Calcineurin inhibitors; MMF-Mycophenolate mofetil; IVIG- Intravenous immunoglobulin; IS- Immunosuppression

### Early vs. late nephritis

We conducted a comparative analysis of early (*≤* 90 days) versus late (> 90 days) adenoviral infections. Early adenoviral infection was significantly more prevalent among cadaveric donors. Fever and gross haematuria were commoner in late infections. No significant differences in biopsy findings and outcomes were noted between the two groups (Table 5).

**Table 5:** Comparison of early-onset and late-onset adenoviral nephritis in renal allograft recipients

	Early adenoviral nephritis (*≤* 90 days) (*n* = 27)	Late adenoviral nephritis (> 90 days) (*n* = 30)	*p*-value
Age	47.7 *±* 13.8	44 *±* 14.9	0.34
Cadaveric donor	20/24 (83.3%)	16/29 (55.1%)	0.029
**Clinical symptoms**			
Fever	13/26 (50%)	24/28(85.7%)	0.005
Gross haematuria	10/27 (37%)	19/30 (63.3%)	0.047
Microscopic haematuria	18/25 (72%)	25/29 (86.2%)	0.196
Dysuria	11/26 (42.3%)	16/29 (55.1%)	0.34
Diarrhoea	8/27 (29.6%)	4/29 (13.7%)	0.14
**Biopsy**			
Granulomas	12/24 (50%)	17/25 (68%)	0.20
Polymorphonuclear infiltrates	15/21 (71.4%)	15/24 (62.5%)	0.52
**Outcomes**			
Reversible graft dysfunction	18/27 (66.6%)	25/30 (83.3%)	0.14
Mortality	3/27 (11.1%)	1/30 (0.03%)	0.25

### Critical appraisal of literature

All reports successfully described patient demographic characteristics, histories, clinical presentations, diagnostic assessments, and treatment interventions (Supplementary Tables 1 and 2). Except for six cases, other reports either did not identify these events or remained ambiguous, indicating areas for improvement in documentation.

### Reversible vs. irreversible graft dysfunction

Irreversible graft dysfunction was not influenced by gender, timing of adenoviral infection (early vs. late), type of donor (cadaveric or live), or the presence or absence of granulomas on biopsy (Table 6). However, patients presenting with dysuria and hematuria, particularly microscopic haematuria, were more likely to achieve complete graft recovery.

**Table 6:** Univariate analysis of factors comparing reversible vs. irreversible graft dysfunction

Variables		Reversible graft dysfunction (*n* = 43)	Irreversible graft dysfunction (*n* = 14)	*p*-value
Gender	Male	29/43 (67.44%)	13/14 (92.86%)	0.061
	Female	14/43 (32.56%)	1/14 (7.14%)	
Early vs. late	Early	18/43 (41.86%)	9/14 (64.29%)	0.144
	Late	25/43 (58.14%)	5/14 (35.71%)	
Donor	Deceased	24/40 (60.00%)	12/13 (92.31%)	0.096
	Live	16/40 (40.00%)	1/13 (7.69%)	
Clinical features	Fever	30/43 (69.77%)	7/14 ()	0.156
	Dysuria	27/43 (62.79%)	0/12 (0.00%)	< 0.001
	Diarrhoea	10/43 (23.26%)	2/13 (15.38%)	0.176
	Macroscopic haematuria	25/43 (58.14%)	4/14 (28.57%)	0.055
	Microscopic haematuria	37/42 (88.10%)	6/12 (50.00%)	0.004
Biopsy findings	Mononuclear infiltrate	24/35 (68.57%)	3/10 (30.00%)	0.077
	Polymorphonuclear	21/43 (48.84%)	9/10 (90.00%)	0.168
	Acute tubular necrosis	20/36 (55.56%)	6/13 (46.15%)	0.581
	Granuloma	20/36 (55.56%)	09/13 (69.23%)	0.47
Viraemia		25/27 (92.59%)	9/9 (100.00%)	0.47
Viruria		25/26 (96.15%)	8/8 (100.00%)	0.837
Reduction in Immunosuppression		39/43 (90.70%)	14/14 (100.00%)	0.237

## Discussion

Adenovirus typically leads to self-limiting illnesses in immunocompetent individuals; however, in immunocompromised hosts, such as those who have undergone stem cell or solid organ transplants, it can precipitate disseminated infections and significantly increase mortality [1–3]. In renal allograft recipients presenting with fever and graft dysfunction, it is imperative to differentiate adenoviral infections from other potential causes, including rejection and other infections [bacterial (*Escherichia coli*,* Klebsiella pneumoniae*, etc.), fungal (*Candida* spp) and viral (BK, Cytomegalovirus)] due to the potential implications for treatment.

Our review indicated that early adenoviral infections are widespread among patients receiving cadaveric/deceased donor kidney transplants, likely attributable to higher levels of immunosuppression associated with these transplants, which may facilitate reactivation. Reactivation of latent adenoviral infections or transmission from the donor is more prevalent during early adenoviral infections, whereas late infections are often primary infections, acquired through the respiratory or gastrointestinal tract [46, 47].

Fever was present in 68% of cases, indicating that the absence of fever does not exclude the possibility of an adenoviral infection. Fever was more common in late-onset adenoviral infection than early infection, possibly due to higher immunosuppression during the early phase. It must also be noted that besides other infections, fever has been commonly reported in graft rejection as well and thus it may be difficult to distinguish adenoviral nephritis from grant rejection while awaiting the results of confirmatory testing [48]. In our SRMA, more than half of the cases exhibited gross haematuria, suggesting possible coexisting haemorrhagic cystitis. Despite the common occurrence of gross haematuria, its absence does not exclude the possibility of adenoviral nephritis, necessitating a high index of suspicion for diagnosis. Haematuria has also been reported in BK viral nephropathy, albeit not as commonly [7].

Radiological findings in adenoviral infections are often unremarkable; however, certain abnormalities may be observed, such as hydronephrosis, pelvicalyceal thickening, hypodense lesions in the renal cortex, and mass-like lesions in the kidney. Hydronephrosis in adenoviral infections can result from ureteral involvement or blood clots in the urine due to concomitant haemorrhagic cystitis. Hypodense lesions with areas of hypoperfusion, which may appear mass-like, have been reported in cases of adenoviral nephritis [14, 27, 29, 30, 34, 42, 43]. Adenovirus is a potent stimulator of both innate and adaptive immunity. Infection with adenovirus can lead to severe tubulointerstitial edema accompanied by necrosis without any involvement of blood vessels. These focal necrotic lesions often resemble other conditions, such as infarcts, abscesses, and post-transplant lymphoproliferative disorders. When necrotizing lesions are observed along with significant inflammation and without evidence of vascular involvement, adenoviral infection should be strongly considered.

Histopathological features such as tubulocentric inflammation, mixed or polymorphonuclear infiltrates, granulomas and inclusion bodies are seen in adenoviral nephritis [7]. Polymorphonuclear infiltrates in biopsy specimens, considered a marker of bacterial infection, were common in adenoviral nephritis. Granulomas were commonly seen in our review, a feature rarely observed in BK nephropathy, which more frequently presents with fibrosis [49]. Surprisingly, necrotizing granulomas, classically described in tuberculosis, were frequently reported in the included cases without any suggestion of tuberculosis co-infection [50]. Vascular involvement in adenoviral infections is uncommon; thus, its presence should prompt further evaluation for antibody-mediated rejection or CMV nephritis [51, 52]. Only one patient in our review demonstrated vascular involvement, and this individual also presented with concomitant thrombotic microangiopathy [12]. Many patients may exhibit interstitial nephritis characterised solely by lymphomonocytic infiltrates, complicating the differentiation from graft rejection.

In those patients where renal biopsy cannot be done, PCR testing for adenovirus in serum and urine can also facilitate diagnosis. Among patients with characteristic biopsy features such as tubulocentric inflammation and granulomas, a positive PCR result in urine or lung specimens can support a presumptive diagnosis of adenovirus. Our review detected viremia in 94% (34/36) of cases, while viruria was observed in 97% (33/34) of instances. It should also be noted that asymptomatic viremia has been documented in approximately 6% of cases within the first year post-transplantation in earlier studies [53].

The management of adenoviral nephritis in the context of renal transplantation primarily revolves around reducing immunosuppression. The use of intravenous immunoglobulin and antiviral agents such as cidofovir, ganciclovir, and ribavirin remains contentious due to the limited literature available [54–56]. Cidofovir’s nephrotoxicity poses significant concerns, while ganciclovir and ribavirin exhibit limited antiviral efficacy against adenovirus [56]. Some authors advocate for low-dose daily cidofovir, which may theoretically minimize nephrotoxicity compared to standard dosing, particularly in cases with urinary tract involvement, where cidofovir is concentrated [57, 58]. A few experts recommend administering cidofovir without probenecid, as this may enhance the concentration of cidofovir within renal tubular epithelial cells [58].

Outcomes for adenoviral nephritis are generally favourable, with predominantly reversible graft dysfunction, rarely leading to chronic changes or graft loss. The presence of dysuria and microscopic haematuria were more commonly associated with the reversibility of graft dysfunction. These could be a chance finding, but it is possible that the presence of these symptoms would have led to early recognition of graft dysfunction and consequent reduction in immunosuppression. Nevertheless, reducing immunosuppression during an adenoviral infection may increase the risk of subsequent rejection [7]. Following the initial reduction of immunosuppression and successful resolution of the infection, immunosuppression can be intensified to ensure optimal patient outcomes.

This review had several limitations. The data was extracted from individual case reports or series with no standardized reporting format. Consequently, data on some variables were not available for all the patients. Also, owing to the small sample size and outcome events, we could not perform a logistic regression analysis to understand predictors of poor outcomes.

In conclusion, adenoviral nephritis is associated with diverse clinical manifestations with differing chronology post-transplantation. Graft dysfunction is associated with reversible interstitial nephritis. Early recognition and differentiation from rejection and other viral infections are crucial for effective management. Key histopathological features and serum or urine PCR testing aid in diagnosis, while balancing immunosuppression is essential to mitigate infection severity and rejection risk. Although outcomes for adenoviral nephritis are generally favourable, careful management is necessary to navigate these complexities. Ongoing research will be vital to enhance understanding and improve outcomes for this vulnerable patient population.

## Electronic Supplementary Material

Below is the link to the electronic supplementary material.

Supplementary Material 1

## Data Availability

No datasets were generated or analysed during the current study.

## References

[1] Kojaoghlanian T, Flomenberg P, Horwitz MS. The impact of adenovirus infection on the immunocompromised host. Rev Med Virol. 2003;13(3):155–71. 12740831 10.1002/rmv.386

[2] Zahradnik JM, Spencer MJ, Porter DD. Adenovirus infection in the immunocompromised patient. Am J Med. 1980;68(5):725–32. 6246799 10.1016/0002-9343(80)90262-4

[3] Shields AF, Hackman RC, Fife KH, Corey L, Meyers JD. Adenovirus infections in patients undergoing bone-marrow transplantation. N Engl J Med. 1985;312(9):529–33. 2982098 10.1056/NEJM198502283120901

[4] Florescu MC, Miles CD, Florescu DF. What do we know about adenovirus in renal transplantation? Nephrol Dial Transplant Off Publ Eur Dial Transpl Assoc -. Eur Ren Assoc. 2013;28(8):2003–10. 10.1093/ndt/gft036 23493328

[5] Watcharananan SP, Avery R, Ingsathit A, Malathum K, Chantratita W, Mavichak V, et al. Adenovirus disease after kidney transplantation: course of infection and outcome in relation to blood viral load and immune recovery. Am J Transpl Off J Am Soc Transpl Am Soc Transpl Surg. 2011;11(6):1308–14. 10.1111/j.1600-6143.2011.03479.x 21449944

[6] Bruminhent J, Worawichawong S, Tongsook C, Pasomsub E, Boongird S, Watcharananan SP. Epidemiology and Outcomes of Early-Onset and Late-Onset Adenovirus Infections in Kidney Transplant Recipients. Open Forum Infect Dis. 2019;6(12):ofz489. 32128332 10.1093/ofid/ofz489 PMC7047955

[7] Jagannathan G, Weins A, Daniel E, Crew RJ, Swanson SJ, Markowitz GS, et al. The pathologic spectrum of adenovirus nephritis in the kidney allograft. Kidney Int. 2023;103(2):378–90. 36436678 10.1016/j.kint.2022.10.025

[8] Hofland CA, Eron LJ, Washecka RM. Hemorrhagic adenovirus cystitis after renal transplantation. Transplant Proc. 2004;36(10):3025–7. 10.1016/j.transproceed.2004.10.090 15686686

[9] Sujeet K, Vasudev B, Desai P, Bellizzi J, Novoa-Takara L, He C, et al. Acute kidney injury requiring dialysis secondary to adenovirus nephritis in renal transplant recipient. Transpl Infect Dis Off J Transpl Soc. 2011;13(2):174–7. 10.1111/j.1399-3062.2010.00577.x 20946204

[10] Rady K, Walters G, Brown M, Talaulikar G. Allograft adenovirus nephritis. Clin Kidney J. 2014;7(3):289–92. 25852891 10.1093/ckj/sfu020 PMC4377743

[11] Friedrichs N, Eis-Hubinger AM, Heim A, Platen E, Zhou H, Buettner R. Acute adenoviral infection of a graft by serotype 35 following renal transplantation. Pathol Res Pract. 2003;199(8):565–70. 14533942 10.1078/0344-0338-00463

[12] Sanathkumar HT, Kurien AA, Raj YT, Fernando EM. Adenovirus-Associated Thrombotic Microangiopathy and Necrotizing Interstitial Nephritis in a Renal Transplant Recipient: A Case Report and Review. Indian J Nephrol. 2021;31(3):314–8. 34376953 10.4103/ijn.IJN_344_19 PMC8330649

[13] Seralathan G, Kurien AA. Adenovirus Interstitial Nephritis: An Unusual Cause for Early Graft Dysfunction. Indian J Nephrol. 2018;28(5):385–8. 30271002 10.4103/ijn.IJN_218_16 PMC6146728

[14] Watanabe M, Kaneko S, Usui J, Takahashi K, Kawanishi K, Takahashi-Kobayashi M, et al. Literature review of allograft adenovirus nephritis and a case presenting as mass lesions in a transplanted kidney without symptoms of urinary tract infection or acute kidney injury. Transpl Infect Dis Off J Transpl Soc. 2021;23(2):e13468. 10.1111/tid.13468 32945064

[15] Lilley CM, Borys E, Picken MM. Adenovirus-Associated Acute Interstitial Nephritis With Graft Survival and Novel Follow-Up Biopsy Findings Including Karyomegaly: A Case Series. Cureus. 2023;15(5):e38452. 37273386 10.7759/cureus.38452 PMC10234624

[16] Fontalvo N, Álvarez H, Osorio W, Garrido D. Primer caso de infección por adenovirus después de un trasplante de riñón en ecuador. Rev Nefrol Diálisis Traspl. 2019;39(3):198–201.

[17] Lum EL, Zuckerman J, Gaynor P, Bunnapradist S. Adenovirus in a Kidney Transplant Recipient. Kidney Med. 2023;5(4):100605. 36915369 10.1016/j.xkme.2023.100605 PMC10006501

[18] Attieh RM, Roach D, Wadei HM, Parikh N, Me HM, Durvasula RV et al. Case Report: Early-Onset Adenovirus Nephritis Without Hemorrhagic Cystitis Following Kidney Transplantation. Transplant Proc. 2024;56(5):1196–9. 10.1016/j.transproceed.2024.05.018 38851958

[19] Fujita Y, Fujishima R, Ueki K, Tsuchimoto A, Matsuda T, Kato M, et al. Allograft adenovirus nephritis accompanied by Crohn’s disease in a kidney transplant recipient: a novel case report. CEN Case Rep. 2023;12(2):215–20. 36399319 10.1007/s13730-022-00756-5 PMC10151298

[20] Pochineni V, Randhawa P, Puttarajappa C. Fever and Gross Hematuria in Kidney Transplant Recipient. Am J Kidney Dis Off J Natl Kidney Found. 2018;72(4):A15–8. 10.1053/j.ajkd.2018.06.009 30244696

[21] An L, Patel P, Ho CH. Five-day fever · elevated creatinine levels · kidney transplant 10 months prior · Dx? J Fam Pract. 2019;68(1):E12–4. 30724910

[22] Alquadan KF, Womer KL, Santos AH, Zeng X, Koratala A. Not all inflammation in a renal allograft is rejection. Clin Case Rep. 2018;6(11):2285–6. 30455940 10.1002/ccr3.1825 PMC6230630

[23] Thorne P, Arroyo JP, Concepcion BP. Fever and Gross Hematuria in a Kidney Transplant Recipient. Kidney360. 2020;1(7):712–3. 35372939 10.34067/KID.0000732020 PMC8815547

[24] Moreira L, Rocha C, Silva J, Silva M, Almeida J, Pedroso M. Adenovirus infection-A rare cause of interstitial nephritis in kidney transplant. Nefrologia. 2019;39(1):106–7. 30097206 10.1016/j.nefro.2018.06.001

[25] Barros Silva GE, Muglia VF, Filho NS, Medeiros de Araújo E, Lages JS, Alves Ferreira TC, et al. Adenovirus pyelonephritis in the late posttransplant period. Kidney Int. 2017;92(2):520. 28709609 10.1016/j.kint.2017.03.016

[26] Veer M, Abdulmassih R, Como J, Min Z, Bhanot N. Adenoviral nephritis in a renal transplant recipient: Case report and literature review. Transpl Infect Dis Off J Transpl Soc. 2017;19(4). 10.1111/tid.12716 28467620

[27] Nanmoku K, Ishikawa N, Kurosawa A, Shimizu T, Kimura T, Miki A, et al. Clinical characteristics and outcomes of adenovirus infection of the urinary tract after renal transplantation. Transpl Infect Dis Off J Transpl Soc. 2016;18(2):234–9. 10.1111/tid.12519 26919131

[28] Park UJ, Hyun SK, Kim HT, Cho WH, Han SY. Successful treatment of disseminated adenovirus infection with ribavirin and intravenous immunoglobulin in an adult renal transplant recipient: a case report. Transpl Proc. 2015;47(3):791–3. 10.1016/j.transproceed.2014.11.054 25891733

[29] Saliba M, Kfoury Assouf H, Abbas S, Abi Hanna P, Kamel G, Barbari A. Adenovirus Infection as a Cause of Fever of Unknown Origin and Allograft Dysfunction in a Kidney Transplant Recipient. Exp Clin Transpl Off J Middle East Soc Organ Transpl. 2019;17(3):411–3. 10.6002/ect.2017.0181 29025381

[30] Ramírez J, Bostock IC, Martin-Onraët A, Calleja S, Sánchez-Cedillo A, Navarro-Vargas LA, et al. Fever, haematuria, and acute graft dysfunction in renal transplant recipients secondary to adenovirus infection: two case reports. Case Rep Nephrol. 2013;2013:195753. 24558620 10.1155/2013/195753 PMC3914224

[31] Parasuraman R, Zhang PL, Samarapungavan D, Rocher L, Koffron A. Severe necrotizing adenovirus tubulointerstitial nephritis in a kidney transplant recipient. Case Rep Transpl. 2013;2013:969186. 10.1155/2013/969186 PMC3771480 24066254

[32] Joyon N, François H, Guettier C, Ferlicot S. [A rare cause of renal graft dysfunction]. Ann Pathol. 2011;31(2):116–8. 21601118 10.1016/j.annpat.2011.02.003

[33] Storsley L, Gibson IW. Adenovirus interstitial nephritis and rejection in an allograft. J Am Soc Nephrol JASN. 2011;22(8):1423–7. 21436288 10.1681/ASN.2010090941 PMC3148696

[34] Varma MC, Kushner YB, Ko DS, Kawai T, Martins PN, Kaur P, et al. Early onset adenovirus infection after simultaneous kidney-pancreas transplant. Am J Transpl Off J Am Soc Transpl Am Soc Transpl Surg. 2011;11(3):623–7. 10.1111/j.1600-6143.2010.03408.x 21342452

[35] Kozlowski T, Nickeleit V, Andreoni K. Donor-transmitted adenovirus infection causing kidney allograft nephritis and graft loss. Transpl Infect Dis Off J Transpl Soc. 2011;13(2):168–73. 10.1111/j.1399-3062.2010.00572.x 20854282

[36] Kolankiewicz LM, Pullman J, Raffeld M, Kopp JB, Glicklich D. Adenovirus nephritis and obstructive uropathy in a renal transplant recipient: case report and literature review. NDT Plus. 2010;3(4):388–92. 25949439 10.1093/ndtplus/sfq024 PMC4421518

[37] Barraclough K, Oliver K, Playford EG, Preston J, Campbell S, Johnson DW, et al. Life-threatening adenovirus infection in a kidney transplant recipient. NDT Plus. 2009;2(3):250–3. 25984003 10.1093/ndtplus/sfp003 PMC4421196

[38] Paula FJ, Neves PDMM, Lazari CS, Ramos RG, Frediani MM, Silva MVA, et al. The Case| Unexplained fever and acute kidney injury in a kidney transplant patient. Kidney Int. 2016;90(6):1391–2. 27884317 10.1016/j.kint.2016.07.003

[39] Hensley JL, Sifri CD, Cathro HP, Lobo P, Sawyer RG, Brayman KL, et al. Adenoviral graft-nephritis: case report and review of the literature. Transpl Int Off J Eur Soc Organ Transpl. 2009;22(6):672–7. 10.1111/j.1432-2277.2009.00838.x 19210749

[40] Gaspert A, Lüthi B, Mueller NJ, Bossart W, Heim A, Wüthrich RP, et al. Subacute allograft failure with dysuria and hematuria in a kidney transplant recipient. Am J Kidney Dis Off J Natl Kidney Found. 2009;54(1):154–8. 10.1053/j.ajkd.2008.11.008 19121556

[41] Alsaad KO, Tobar A, Belanger E, Ahmad M, Cattran DC, Herzenberg AM. Late-onset acute haemorrhagic necrotizing granulomatous adenovirus tubulointerstitial nephritis in a renal allograft. Nephrol Dial Transplant Off Publ Eur Dial Transpl Assoc -. Eur Ren Assoc. 2007;22(4):1257–60. 10.1093/ndt/gfl843 17267541

[42] Rosario RF, Kimbrough RC, Van Buren DH, Laski ME. Fatal adenovirus serotype-5 in a deceased-donor renal transplant recipient. Transpl Infect Dis Off J Transpl Soc. 2006;8(1):54–7. 10.1111/j.1399-3062.2006.00137.x 16623822

[43] Lim AKH, Parsons S, Ierino F. Adenovirus tubulointerstitial nephritis presenting as a renal allograft space occupying lesion. Am J Transpl Off J Am Soc Transpl Am Soc Transpl Surg. 2005;5(8):2062–6. 10.1111/j.1600-6143.2005.00945.x 15996261

[44] Asim M, Chong-Lopez A, Nickeleit V. Adenovirus infection of a renal allograft. Am J Kidney Dis Off J Natl Kidney Found. 2003;41(3):696–701. 10.1053/ajkd.2003.50133 12612996

[45] Lachiewicz AM, Cianciolo R, Miller MB, Derebail VK. Adenovirus causing fever, upper respiratory infection, and allograft nephritis complicated by persistent asymptomatic viremia. Transpl Infect Dis Off J Transpl Soc. 2014;16(4):648–52. 10.1111/tid.12248 24966111

[46] Ac S, Cl XLESAL et al. E, S P,. Human Adenovirus 11 in 2 Renal Transplant Recipients: Suspected Donor-Derived Infection. Open Forum Infect Dis [Internet]. 2021 Feb 25 [cited 2024 Oct 5];8(3). https://pubmed.ncbi.nlm.nih.gov/34386544/10.1093/ofid/ofab092 PMC8355461 34386544

[47] Pettengill MA, Babu TM, Prasad P, Chuang S, Drage MG, Menegus M, et al. Probable Donor-Derived Human Adenovirus Type 34 Infection in 2 Kidney Transplant Recipients From the Same Donor. Open Forum Infect Dis. 2019;6(3):ofy354. 30882008 10.1093/ofid/ofy354 PMC6411205

[48] Toogood GJ, Roake JA, Morris PJ. The relationship between fever and acute rejection or infection following renal transplantation in the cyclosporin era. Clin Transpl. 1994;8(4):373–7. 7949542

[49] Jamboti JS. BK virus nephropathy in renal transplant recipients. Nephrol Carlton Vic. 2016;21(8):647–54. 10.1111/nep.12728 26780694

[50] Ab F, Cl E, Te R, Wj C, Sm AC. M. Renal allograft granulomatous interstitial nephritis: observations of an uncommon injury pattern in 22 transplant recipients. Clin Kidney J [Internet]. 2017 Apr [cited 2024 Oct 5];10(2). https://pubmed.ncbi.nlm.nih.gov/28396741/10.1093/ckj/sfw117 PMC5381240 28396741

[51] Swanson KJ, Djamali A, Jorgenson MR, Misch EA, Ghaffar A, Zhong W, et al. Cytomegalovirus nephritis in kidney transplant recipients: Epidemiology and outcomes of an uncommon diagnosis. Transpl Infect Dis Off J Transpl Soc. 2021;23(5):e13702. 10.1111/tid.13702 34324253

[52] Najafian B, Fogo AB, Lusco MA, Alpers CE. AJKD Atlas of Renal Pathology: acute antibody-mediated rejection. Am J Kidney Dis Off J Natl Kidney Found. 2015;66(5):e39–40. 10.1053/j.ajkd.2015.08.009 26498422

[53] Humar A, Kumar D, Mazzulli T, Razonable RR, Moussa G, Paya CV, et al. A surveillance study of adenovirus infection in adult solid organ transplant recipients. Am J Transpl Off J Am Soc Transpl Am Soc Transpl Surg. 2005;5(10):2555–9. 10.1111/j.1600-6143.2005.01033.x 16162207

[54] Kodama E, Shigeta S, Suzuki T, De Clercq E. Application of a gastric cancer cell line (MKN-28) for anti-adenovirus screening using the MTT method. Antiviral Res. 1996;31(3):159–64. 8811200 10.1016/0166-3542(96)06966-5

[55] Leruez-Ville M, Minard V, Lacaille F, Buzyn A, Abachin E, Blanche S, et al. Real-time blood plasma polymerase chain reaction for management of disseminated adenovirus infection. Clin Infect Dis Off Publ Infect Dis Soc Am. 2004;38(1):45–52. 10.1086/380450 14679447

[56] M B, S M, S S, E DC. Selective inhibitory effect of (S)-9-(3-hydroxy-2-phosphonylmethoxypropyl)adenine and 2’-nor-cyclic GMP on adenovirus replication in vitro. Antimicrob Agents Chemother [Internet]. 1987 Feb [cited 2024 Oct 5];31(2). https://pubmed.ncbi.nlm.nih.gov/3566256/10.1128/aac.31.2.337 PMC174721 3566256

[57] Lamoth F, Pascual M, Erard V, Venetz JP, Nseir G, Meylan P. Low-dose cidofovir for the treatment of polyomavirus-associated nephropathy: two case reports and review of the literature. Antivir Ther. 2008;13(8):1001–9. 19195325

[58] Momper JD, Zhao Y, Shapiro R, Schonder KS, Gao Y, Randhawa PS, et al. Pharmacokinetics of low-dose cidofovir in kidney transplant recipients with BK virus infection. Transpl Infect Dis Off J Transpl Soc. 2013;15(1):34–41. 10.1111/tid.12014 PMC3654813 23025519

