# Supplementary material for: Adenovirus nephritis in adult kidney allograft recipients: a systematic review of literature
Source: Infection. 2025 Jan 2;53(1):25–37. doi: 10.1007/s15010-024-02455-y (PMC11825578; doi:10.1007/s15010-024-02455-y)
Supplement: Supplementary file 1 — Supplementary Material 1 [file 15010_2024_2455_MOESM1_ESM.docx]

Supplementary Table 1: JBI checklist questions for critical appraisal of case reports

| JBI case report questions |
| --- |
| 1. Were the patient’s demographic characteristics clearly described? |
| 2. Was the patient’s history clearly described and presented as a timeline? |
| 3. Was the current clinical condition of the patient on presentation clearly described? |
| 4. Were diagnostic tests or assessment methods and the results clearly described? |
| 5. Was the intervention(s) or treatment procedure(s) clearly described? |
| 6. Was the post-intervention clinical condition clearly described? |
| 7. Were adverse events (harms) or unanticipated events identified and described? |

Supplementary Table 2: Critical Appraisal of the included cases using the JBI checklist

| **Sn** | **Authors** | **Demography** | **History** | **Presentation** | **Diagnosis** | **Treatment** | **Follow-up** | **Adverse event** |
| --- | --- | --- | --- | --- | --- | --- | --- | --- |
| 1 | Sujeet et al | Yes | Yes | Yes | Yes | Yes | Yes | Yes |
| 2 | Rady et al | Yes | Yes | Yes | Yes | Yes | Yes | Not applicable |
| 3 | Friedrichs et al | Yes | Yes | Yes | Yes | Yes | Yes | Not applicable |
| 4 | Harshavardhan et al | Yes | Yes | Yes | Yes | Yes | Yes | Not applicable |
| 5 | Seralathan et al | Yes | Yes | Yes | Yes | Yes | Yes | Not applicable |
| 6 | Watanabe et al | Yes | Yes | Yes | Yes | Yes | Yes | Not applicable |
| 7 | Cullen et al | Yes | Unclear | Yes | Yes | Yes | Yes | Not applicable |
| 8 | Cullen et al | Yes | Unclear | Yes | Yes | Yes | Yes | Yes |
| 9 | Cullen et al | Yes | Unclear | Yes | Yes | Yes | Yes | Yes |
| 10 | Fontalvo et al | Yes | Yes | Yes | Yes | Yes | Yes | Not applicable |
| 11 | Lum et al | Yes | Yes | Yes | Yes | Yes | Yes | Yes |
| 12 | Bruminhent et al | Yes | Yes | Yes | Yes | Yes | Yes | Unclear |
| 13 | Bruminhent et al | Yes | Yes | Yes | Yes | Yes | Yes | Unclear |
| 14 | Attieh et al | Yes | Yes | Yes | Yes | Yes | Yes | Unclear |
| 15 | Jagannatham et al | Yes | Yes | Yes | Yes | Yes | Yes | Not applicable |
| 16 | Jagannatham et al | Yes | Yes | Yes | Yes | Yes | Yes | Unclear |
| 17 | Jagannatham et al | Yes | Yes | Yes | Yes | Yes | Yes | Unclear |
| 18 | Jagannatham et al | Yes | Yes | Yes | Yes | Yes | Yes | Not applicable |
| 19 | Jagannatham et al | Yes | Yes | Yes | Yes | Yes | Yes | Not applicable |
| 20 | Jagannatham et al | Yes | Yes | Yes | Yes | Yes | Yes | Not applicable |
| 21 | Jagannatham et al | Yes | Yes | Yes | Yes | Yes | Yes | Not applicable |
| 22 | Jagannatham et al | Yes | Yes | Yes | Yes | Yes | Yes | Unclear |
| 23 | Jagannatham et al | Yes | Yes | Yes | Yes | Yes | Yes | Not applicable |
| 24 | Jagannatham et al | Yes | Yes | Yes | Yes | Yes | Yes | Not applicable |
| 25 | Jagannatham et al | Yes | Yes | Yes | Yes | Yes | Yes | Not applicable |
| 26 | Fujita et al | Yes | Yes | Yes | Yes | Yes | Yes | Not applicable |
| 27 | Pochineni et al | Yes | Yes | Yes | Yes | Yes | Yes | Unclear |
| 28 | Lucia et al | Yes | No | Yes | Unclear | Yes | Yes | Not applicable |
| 29 | Alquadan et al | Yes | Yes | Yes | Yes | Yes | Yes | Not applicable |
| 30 | Thorne et al | Yes | Yes | Yes | Yes | Yes | Yes | Not applicable |
| 31 | Moreira et al | Yes | Yes | Yes | Yes | Yes | Yes | Not applicable |
| 32 | Silva et al | Yes | Unclear | Yes | Yes | Yes | Yes | Not applicable |
| 33 | Veer et al | Yes | Yes | Yes | Yes | Yes | Yes | Not applicable |
| 34 | Nanmoku et al | Yes | Unclear | Yes | Yes | Yes | Yes | Not applicable |
| 35 | Nanmoku et al | Yes | Unclear | Yes | Yes | Yes | Yes | Not applicable |
| 36 | Nanmoku et al | Yes | Unclear | Yes | Yes | Yes | Yes | Not applicable |
| 37 | Nanmoku et al | Yes | Unclear | Yes | Yes | Yes | Yes | Not applicable |
| 38 | Nanmoku et al | Yes | Unclear | Yes | Yes | Yes | Yes | Unclear |
| 39 | Park et al | Yes | Yes | Yes | Yes | Yes | Yes | Unclear |
| 40 | Saliba et al | Yes | Yes | Yes | Yes | Yes | Yes | Not applicable |
| 41 | Ramirez et al | Yes | Yes | Yes | Yes | Yes | Yes | Not applicable |
| 42 | Parasuraman et al | Yes | Yes | Yes | Yes | Yes | Yes | Yes |
| 43 | Joyon et al | Yes | Yes | Yes | Yes | Yes | Yes | Unclear |
| 44 | Storsley et al | Yes | Yes | Yes | Yes | Yes | Yes | Not applicable |
| 45 | Varma et al | Yes | Yes | Yes | Yes | Yes | Yes | Not applicable |
| 46 | Kozlowski et al | Yes | Yes | Yes | Yes | Unclear | Yes | Not applicable |
| 47 | Kozlowski et al | Yes | Yes | Yes | Yes | Unclear | Yes | Not applicable |
| 48 | Kolankiewicz et al | Yes | Yes | Yes | Yes | Yes | Yes | Not applicable |
| 49 | Barraclough et al | Yes | Yes | Yes | Yes | Yes | Yes | Yes |
| 50 | Paula et al | Yes | Yes | Yes | Yes | Yes | Yes | Unclear |
| 51 | Hensley et al | Yes | Yes | Yes | Yes | Yes | Yes | Not applicable |
| 52 | Gaspert et al | Yes | Yes | Yes | Yes | Yes | Yes | Not applicable |
| 53 | Alsaad et al | Yes | Yes | Yes | Yes | Yes | Yes | Unclear |
| 54 | Rosario et al | Yes | Yes | Yes | Unclear | Yes | Yes | Not applicable |
| 55 | Lim et al | Yes | Yes | Yes | Yes | Yes | Yes | Not applicable |
| 56 | Asim et al | Yes | Yes | Yes | Yes | Yes | Yes | Not applicable |
| 57 | Lachiewicz et al | Yes | Yes | Yes | Yes | Yes | Yes | Unclear |
